# Data-driven cluster analysis on the association of aging, obesity and insulin resistance with new-onset diabetes in Chinese adults: a multicenter retrospective cohort study

**DOI:** 10.3389/fmed.2025.1640017

**Published:** 2025-07-30

**Authors:** Yazhi Wang, Mingkang Zhang, Peng Wang

**Affiliations:** ^1^The Second School of Clinical Medicine, Lanzhou University, Lanzhou, Gansu, China; ^2^The School of Pharmacy, Lanzhou University, Lanzhou, Gansu, China; ^3^Department of Pharmacy, The 987th Hospital of Joint Logistics Support Force of People’s Liberation Army, Baoji, Shaanxi, China

**Keywords:** type 2 diabetes mellitus, cluster analysis, aging, obesity, insulin resistance

## Abstract

**Background:**

Type 2 diabetes mellitus (T2DM) is an endocrine and metabolic disorder that can lead to multi-organ damage and dysfunction, imposing significant financial burden on national healthcare systems. Currently, the early identification of high-risk individuals and the prevention of T2DM remain major challenges for clinicians. This study aimed to use easily obtainable clinical indicators to perform cluster analysis on healthy individuals, in order to accurately identify high-risk population requiring early intervention.

**Methods:**

This study was a multicenter retrospective cohort study with a median follow-up period of 3 years. A total of 12,607 Chinese adult individuals without diabetes at baseline were included. The K-means clustering algorithm was applied to five standardized indicators: age, body mass index (BMI), fasting blood glucose (FBG), triglycerides (TG), and HDL-C (high-density lipoprotein cholesterol). After clustering, multivariate Cox proportional hazards regression analysis was used to evaluate and compare the risk of diabetes incidence among different clusters.

**Results:**

The study population comprising 12,607 subjects was clustered into four distinct groups: Cluster 1 (metabolic health cluster), Cluster 2 (low HDL-C cluster), Cluster 3 (old age and mild metabolic disorder cluster), and Cluster 4 (severe obesity and insulin resistance cluster). The proportional distributions of each cluster were 37.95, 29.99, 24.95, and 7.11%, respectively. The clinical characteristics and diabetes incidence risks varied significantly among the four clusters. Cluster 4 exhibited the highest diabetes incidence rate, followed by Cluster 3, Cluster 2, and Cluster 1. In all models adjusted for covariates, the diabetes incidence rates in Cluster 3 and Cluster 4 were significantly higher than those in Cluster 1 and Cluster 2. However, no significant difference was observed between Cluster 3 and Cluster 4.

**Conclusion:**

Cluster-based analyses can effectively identify individuals at high risk of diabetes in the normal population. These high-risk groups (clusters 3 and 4) are often associated with aging, obesity, and insulin resistance (IR), necessitating early and targeted interventions.

## 1 Introduction

Type 2 diabetes mellitus (T2DM) is an endocrine and metabolic disorder characterized primarily by insulin resistance (IR) and insufficient insulin secretion (1). According to data from the International Diabetes Federation (IDF), approximately 537 million people worldwide were living with diabetes mellitus (DM) in 2021. By 2045, this number is projected to rise to 783 million (2). Chronic hyperglycemia not only leads to multi-organ damage and dysfunction, including the kidneys, retina, liver, and cardiovascular system, but also contributes to high mortality rates, imposing significant psychological and physiological burdens on individuals (3). Therefore, timely identification of high-risk population for T2DM, screening for risk factors, and implementing early intervention and management are crucial to mitigating its adverse impacts on individuals and healthcare systems.

Currently, the diagnosis of DM still primarily relies on blood glucose, a single metabolic marker. However, due to the combined influence of genetic, environmental, and lifestyle factors, DM exhibits significant heterogeneity, particularly in T2DM, which accounts for over 90% of all DM cases (1). In fact, multiple factors contribute to the onset and progression of DM, including abnormal pancreatic islet development, impaired islet function, autoimmunity, inflammation, reduced insulin sensitivity, and decreased incretin activity, among others (4). The predominant role of a single factor or the synergistic effects of multiple factors can lead to substantial phenotypic variability among individuals with DM (5). Consequently, the approach of focusing solely on blood glucose control is insufficient for preventing DM in the general population.

Clustering analysis may offer a potential solution to the aforementioned challenges. As an unsupervised machine learning algorithm, it categorizes study subjects into distinct phenotypes based on the similarity of input features (6). In 2018, Ahlqvist et al. (7) utilized clustering analysis on a newly diagnosed DM cohort, incorporating variables such as glutamic acid decarboxylase antibodies (GADA), age, body mass index (BMI), glycated hemoglobin (HbA1c), HOMA2-estimated insulin resistance (HOMA2-IR), and HOMA2-estimated beta-cell function (HOMA2-β). Their analysis identified five subtypes with markedly different clinical phenotypes and metabolic characteristics. Similarly, Ye et al. (8) employed clustering analysis on metabolic parameters, including age, BMI, HbA1c, and triglycerides (TG), to develop and validate a novel classification for metabolic dysfunction-associated fatty liver disease (MAFLD) in Chinese and United Kingdom (UK) cohorts. This approach enabled more accurate identification of DM, coronary heart disease, and stroke risks across different subtypes. Furthermore, multiple studies have demonstrated that the results of clustering analysis maintain a certain degree of robustness even after years of follow-up (9, 10). These findings underscore that clustering analysis could serve as a powerful tool for precision medicine in disease management.

Previous studies have extensively explored risk factors for T2DM in general populations but have overlooked the clinical manifestations, pathophysiological characteristics, and genetic features of specific subgroups. This oversight may hinder effective prevention and management of T2DM (11–13). Additionally, key parameters in DM assessment, such as GADA and C-peptide, are rarely evaluated in clinical practice or epidemiological surveys, limiting their widespread application. Currently, clinical research utilizing clustering analysis to predict T2DM onset remains limited. For these reasons, this study selected five easily accessible indicators in epidemiological screening [age, BMI, fasting blood glucose (FBG), TG, and high-density lipoprotein cholesterol (HDL-C)] to conduct a data-driven clustering analysis in a large Chinese cohort. The aim was to identify clusters with distinct metabolic profiles and compare diabetes incidence rates among these clusters, thereby identifying high-risk populations requiring early intervention prior to diabetes onset.

## 2 Materials and methods

### 2.1 Study design and participants

The data used in this study were derived from a public, non-profit computerized database established by China Rich Healthcare. Initially compiled and uploaded by Chen et al. (14) to the “DATADRYAD” website, the dataset is openly accessible to researchers.^1^ This database encompasses 32 medical institutions across 11 cities in China, including Shanghai, Beijing, Guangzhou, Shenzhen, Chengdu, Nanjing, Wuhan, Hefei, Suzhou, Changzhou, and Nantong. Each participant underwent at least two routine health check-ups between 2010 and 2016 (*n* = 685,277).

**FIGURE 1:**
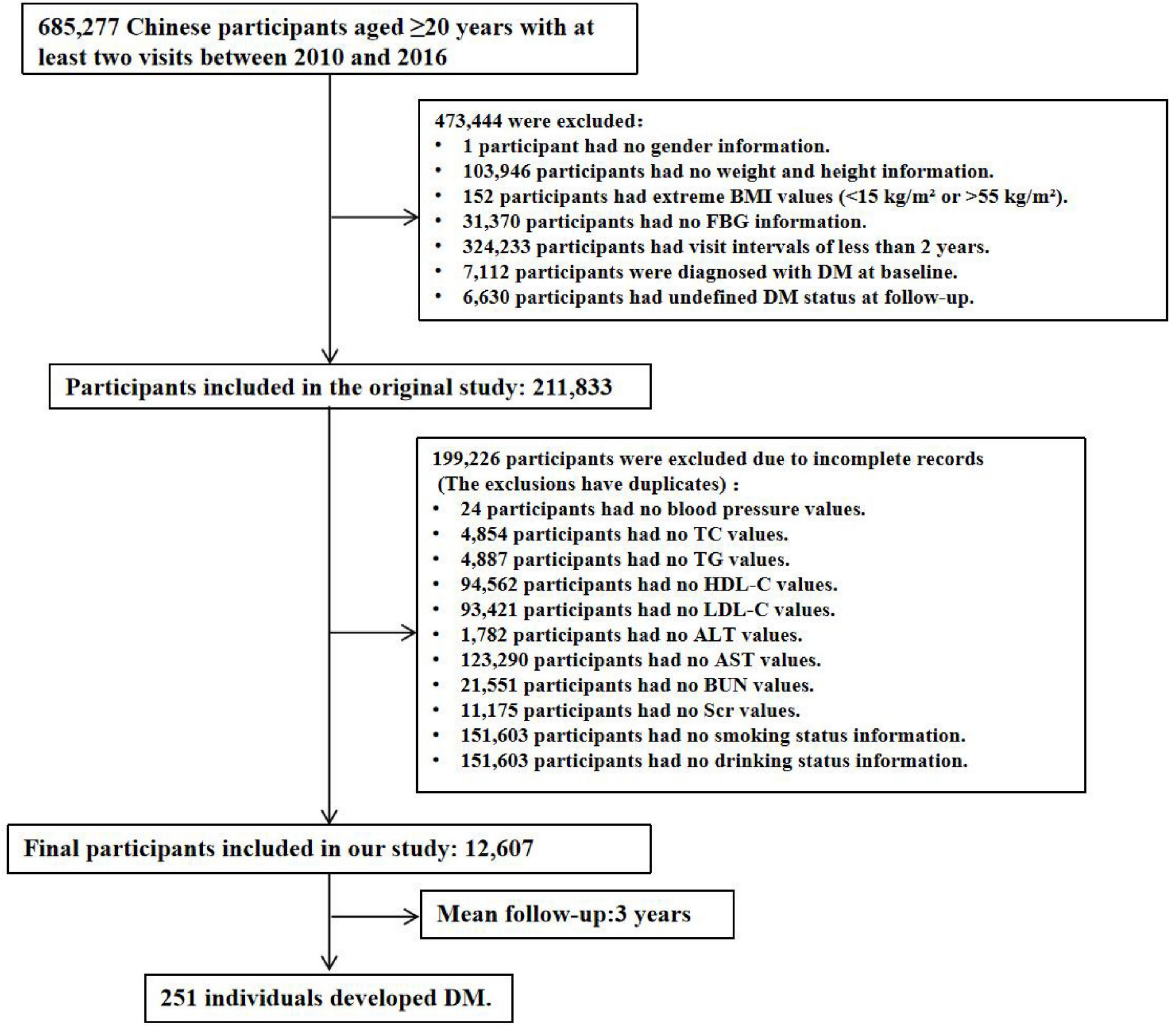
Flowchart of the study population.

During the data compilation process, the following exclusions were applied: missing baseline weight or height (*n* = 103,946), absence of gender information (*n* = 1), missing baseline FBG values (*n* = 31,370), extreme BMI values (< 15 kg/m^2^ or > 55 kg/m^2^, *n* = 152), follow-up intervals of less than 2 years (*n* = 324,233), a history of T2DM at baseline (2,997 participants self-reported a diagnosis, and 4,115 participants were diagnosed based on FPG ≥ 7.0 mmol/L), and participants whose diabetes status remained undetermined at the end of follow-up (*n* = 6,630). After these exclusions, 211,833 participants were initially included. For this study, we further excluded participants with missing baseline variables, resulting in a final cohort of 12,607 participants, as illustrated in Figure 1. This study was conducted in accordance with the Declaration of Helsinki and was approved by the Ethics Committee of the 987th Hospital of the Joint Logistics Support Force of the People’s Liberation Army (Approval No. 2025A-187). Since the database used was publicly available and all participant identities were anonymized, the requirement for informed consent was waived.

### 2.2 Collection, assessment and measurement of covariates

The following clinical information was included in this study. (1) Demographic Information: This encompassed gender (male or female), age, smoking history (current smoker, ever smoker, or never smoker), alcohol consumption history (current drinker, ever drinker, or never drinker), and family history of diabetes (yes or no). These details were collected, recorded, and measured by trained professionals using standardized questionnaires. (2) Anthropometric Measurements: Height, weight, and blood pressure were measured by trained staff. Participants were required to wear lightweight clothing and no shoes during height and weight measurements, which were recorded to the nearest 0.1 cm and 0.1 kg, respectively. BMI was calculated as weight (kg) divided by height squared (m^2^). Blood pressure was measured using the standard mercury sphygmomanometer. (3) Laboratory Indicators: Fasting venous blood samples were collected from participants after at least 10 h of fasting during each visit. FBG, TG, total cholesterol (TC), low-density lipoprotein cholesterol (LDL-C), HDL-C, alanine aminotransferase (ALT), aspartate aminotransferase (AST), blood urea nitrogen (BUN), and serum creatinine (Scr) were measured using the Beckman 5,800 automated analyzer. Standardized procedures were implemented across all analytical equipment to ensure consistency in measurements and parameters. (4) Derived Parameters: To assess the degree of IR, the following indices were calculated. Triglyceride–glucose (TyG) index: TyG = Ln [TG (mg/dL) × FBG (mg/dL)/2] (15). Atherogenic Index of Plasma (AIP): AIP = Log10 [TG (mmol/L)/HDL-C (mmol/L)] (16). Non-HDL-C: Non-HDL-C = TC (mmol/L)—HDL-C (mmol/L) (17). Estimated glomerular filtration rate (eGFR): eGFR was calculated using the CKD-EPI formula (18).

### 2.3 Study outcomes

The outcome event was defined as the occurrence of new-onset diabetes in participants. Diabetes was identified during follow-up if the participant had the FBG level ≥ 7.0 mmol/L and/or self-reported a diagnosis of diabetes (19). The follow-up period ended either on the date of the first occurrence of the outcome event or on the date of the last visit, whichever came first.

### 2.4 Cluster analysis

The clustering analysis for diabetes was performed using the K-means algorithm in R software. Prior to clustering, the five clustering variables (age, BMI, FBG, TG, and HDL-C) were standardized using the Z-score method to eliminate differences in scale and numerical ranges among the variables (20). Subsequently, clustering analysis was conducted on the standardized variables (with a mean of 0 and a standard deviation of 1). In the K-means algorithm, determining the optimal number of clusters (K) is crucial. Based on criteria outlined in previous studies and supported by the elbow method and silhouette measure in TwoStep clustering method (Supplementary Figure S1 and Supplementary Table S1), four clusters were identified as optimal (21, 22). The TwoStep clustering method was applied using log-likelihood as the distance measure and Schwarz’s Bayesian information criterion (BIC) to determine the optimal number of clusters (ranging from 2 to 15). Furthermore, based on the optimal cluster number (*K* = 4) determined above, hierarchical clustering was additionally performed to validate the stability of the k-means clustering results (Supplementary Tables S2, S3). Radar charts were generated for each cluster using Z-scores, which were calculated by adjusting the mean values of the variables within each cluster to the cohort mean and standard deviation.

### 2.5 Statistical analysis

Continuous variables were presented as mean ± standard deviation. Comparisons between two groups were performed using independent samples *t*-tests, while comparisons among multiple groups were conducted using one-way analysis of variance (ANOVA) followed by the least significant difference (LSD) method for *post hoc* pairwise comparisons. Categorical variables were expressed as frequencies (percentages). Comparisons between two or more groups were performed using chi-square tests. If significant differences were observed among multiple groups, *post hoc* pairwise comparisons were conducted with Bonferroni correction. The cumulative risk of diabetes incidence across clusters was compared and analyzed using Kaplan-Meier curves and log-rank tests. To assess the risk of diabetes occurrence in different clusters, multivariate Cox proportional hazards regression analysis was employed to compare the hazard ratios [HRs, 95% confidence intervals (CIs)] of diabetes incidence among the four clusters. To control for confounding variables, three models with progressively adjusted covariates were constructed. Model 1 adjusted for gender, model 2 adjusted for gender, smoking history, alcohol consumption history, and family history of diabetes, and model 3 further adjusted for SBP, DBP, TC, non-HDL-C, LDL-C, ALT, AST, BUN, Scr, and eGFR in addition to the variables in model 2. Statistical analyses were performed using SPSS 27.0 (IBM Corp., Armonk, NY, United States) and R version 4.3.1 (R Foundation for Statistical Computing, Vienna, Austria). A *P*-value < 0.05 was considered statistically significant.

## 3 Results

### 3.1 Basic characteristics of the population

Table 1 summarized the clinical characteristics of the study population, stratified by those who developed new-onset diabetes and those who did not. Over a median follow-up period of 3 years, 251 out of the 12,607 adult participants developed diabetes, comprising 203 males and 48 females. Compared to the non-diabetes group (all *P* < 0.01), participants with new-onset diabetes were older and exhibited higher body weight, BMI, blood pressure (SBP and DBP), blood glucose levels (FBG and FBG at the final visit), and lipid-related parameters (TC, TG, LDL-C, non-HDL-C, TyG index, and AIP). Regarding renal and hepatic function, the new-onset diabetes group had significantly higher levels of BUN, ALT, and AST, but lower eGFR compared to the non-diabetes group (all *P* < 0.001). Additionally, the new-onset diabetes group had higher rates of smoking, alcohol consumption, and the family history of diabetes (all *P* < 0.01). The proportion of individuals with FBG levels between 5.6 and 6.9 mmol/L was also significantly higher in new-onset diabetes population compared to the non-diabetic population (*P* < 0.001).

**TABLE 1:** Basic clinical characteristics of the study population.

Variables	Overall (*n* = 12,607)	Without new-onset DM (*n* = 12,356)	With new-onset DM (*n* = 251)	*P*-value
Age (years)	41.90 ± 11.46	41.67 ± 11.35	53.21 ± 10.91	< 0.001
Gender n, (%)				< 0.001
Male	8,456 (67.1)	8,253 (66.8)	203 (80.9)	
Female	4,151 (32.9)	4,103 (33.2)	48 (19.1)	
Height (cm)	167.78 ± 8.13	167.76 ± 8.13	168.63 ± 8.02	0.093
Weight (kg)	66.70 ± 12.09	66.54 ± 12.04	74.52 ± 11.93	< 0.001
BMI (kg/m^2^)	23.59 ± 3.29	23.54 ± 3.27	26.11 ± 3.17	< 0.001
SBP (mmHg)	119.00 ± 15.26	118.81 ± 15.16	128.65 ± 17.30	< 0.001
DBP (mmHg)	74.74 ± 10.41	74.62 ± 10.36	80.67 ± 11.11	< 0.001
FBG (mmol/L)	5.00 ± 0.61	4.98 ± 0.60	5.96 ± 0.66	< 0.001
FBG_5.6–6.9__*mmol/L*_ n, (%)	1,951 (15.5)	1,775 (14.4)	176 (70.1)	< 0.001
FBG of final visit (mmol/L)	5.19 ± 0.61	5.15 ± 0.49	7.48 ± 1.24	< 0.001
TC(mmol/L)	4.76 ± 0.89	4.76 ± 0.89	5.08 ± 0.86	< 0.001
TG(mmol/L)	1.45 ± 1.12	1.44 ± 1.10	2.28 ± 1.53	< 0.001
HDL-C (mmol/L)	1.35 ± 0.31	1.35 ± 0.30	1.28 ± 0.65	< 0.001
LDL-C (mmol/L)	2.73 ± 0.69	2.73 ± 0.69	2.87 ± 0.68	0.001
Non-HDL-C (mmol/L)	3.80 ± 1.11	3.41 ± 0.86	3.80 ± 1.11	< 0.001
TyG	8.47 ± 0.63	8.46 ± 0.62	9.10 ± 0.62	< 0.001
AIP	–0.04 ± 0.30	–0.05 ± 0.30	0.19 ± 0.31	< 0.001
BUN (mmol/L)	4.75 ± 1.18	4.74 ± 1.17	5.20 ± 1.42	< 0.001
Scr (μmol/L)	73.59 ± 15.19	73.57 ± 15.17	74.40 ± 16.02	0.399
eGFR (mL/min/1.73 m^2^)	102.69 ± 14.02	102.83 ± 13.97	95.72 ± 14.44	< 0.001
ALT (U/L)	25.95 ± 22.18	25.73 ± 22.06	36.47 ± 25.30	< 0.001
AST (U/L)	25.09 ± 10.67	25.00 ± 10.56	29.62 ± 14.76	< 0.001
Smoking status n, (%)				< 0.001
Current smoker	2,524 (20.0)	2,433 (19.7)	91 (36.2)	
Ever smoker	563 (4.5)	543 (4.4)	20 (8.0)	
Never smoker	9,520 (75.5)	9,380 (75.9)	140 (55.8)	
Drinking status n, (%)				0.005
Current drinker	377 (3.0)	361 (2.9)	16 (6.4)	
Ever drinker	2,541 (20.1)	2,487 (20.1)	54 (21.5)	
ever drinker	9,689 (76.8)	9,508 (77.0)	181 (72.1)	
Family history of diabetes n, (%)				< 0.001
Yes	778 (6.2)	748 (6.1)	30 (12.0)	
No	11,829 (93.8)	11,608 (93.9)	221 (88.0)	
Year of follow-up	3.00 ± 0.88	3.00 ± 0.88	3.23 ± 0.98	< 0.001

Values are means ± SD for continuous variables. Values are frequency counts and percentages for categorical variables. *P*-value was calculated by the *t*-test for continuous variables and chi-square tests were performed for categorical variables. *P* < 0.05 was considered significant. DM, diabetes mellitus; BMI, body mass index; SBP, systolic blood pressure; DBP, diastolic blood pressure; FBG, fasting blood glucose; TC, total cholesterol; TG, triglyceride; HDL-C, high-density lipoprotein cholesterol; LDL-C, low-density lipoprotein cholesterol; TyG, triglyceride-glucose index; AIP, atherogenic index of plasma; BUN, blood urea nitrogen; Scr, creatinine; eGFR, estimated glomerular filtration rate; ALT, alanine transferase; AST, aspartate transferase.

### 3.2 Clinical characteristics of the four clusters

**FIGURE 2:**
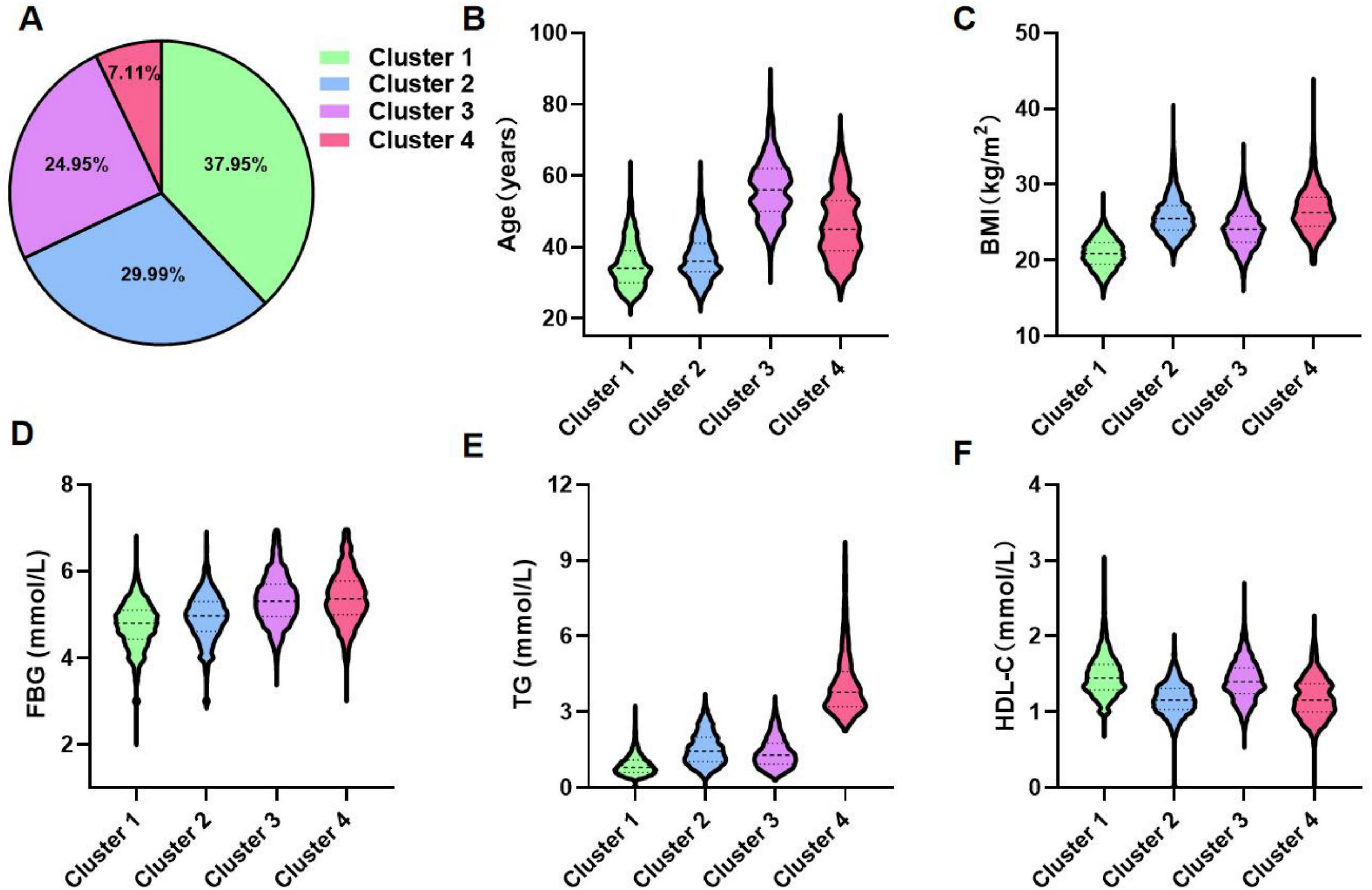
Distribution and clinical features of clusters. **(A)** Proportional distribution of 12,607 participants. **(B–F)** Characteristics of each cluster regarding age, BMI, FBG, TG, and HDL-C. Cluster 1: Metabolic health cluster; Cluster 2: Low HDL-C cluster; Cluster 3: Old age and mild metabolic disorder cluster; Cluster 4: Severe obesity and insulin resistance cluster.

Using the K-means clustering algorithm, the 12,607 participants were categorized into four distinct clusters. Details regarding the cluster centers, which can be used for the stratification of different clusters, were provided in Supplementary Table S4. Figure 2 illustrated the clinical characteristics of the four clusters across the clustering variables. Cluster 1 (*n* = 4,784, 37.95%): This cluster had the youngest participants, with a mean age of 35.22 years. It exhibited the lowest levels of BMI, FBG, and TG, along with the highest levels of HDL-C. Given its optimal metabolic profile, this cluster was labeled the metabolic health cluster (MHC). Cluster 2 (*n* = 3,781, 29.99%): This cluster demonstrated the relatively favorable metabolic state, with a mean age of 37.25 years, lower FBG and TG levels, and moderate BMI. However, it had the lowest HDL-C levels among the clusters, leading to its designation as the low HDL-C cluster (LHC). Cluster 3 (*n* = 3,146, 24.95%): This cluster exhibited the moderate metabolic profile. It had the oldest participants, with a mean age of 56.41 years, along with relatively higher BMI and FBG levels and moderate HDL-C levels. It was named the old age and mild metabolic disorder cluster (OMDC). Cluster 4 (*n* = 896, 7.11%): This cluster displayed the poorest metabolic state. Participants were relatively older, with a mean age of 46.22 years, and had the highest levels of BMI, FBG, and TG, coupled with relatively low HDL-C levels. Consequently, it was designated the severe obesity and insulin resistance cluster (SOIRC).

### 3.3 Basic information and biochemical parameters characteristics of each cluster

**FIGURE 3:**
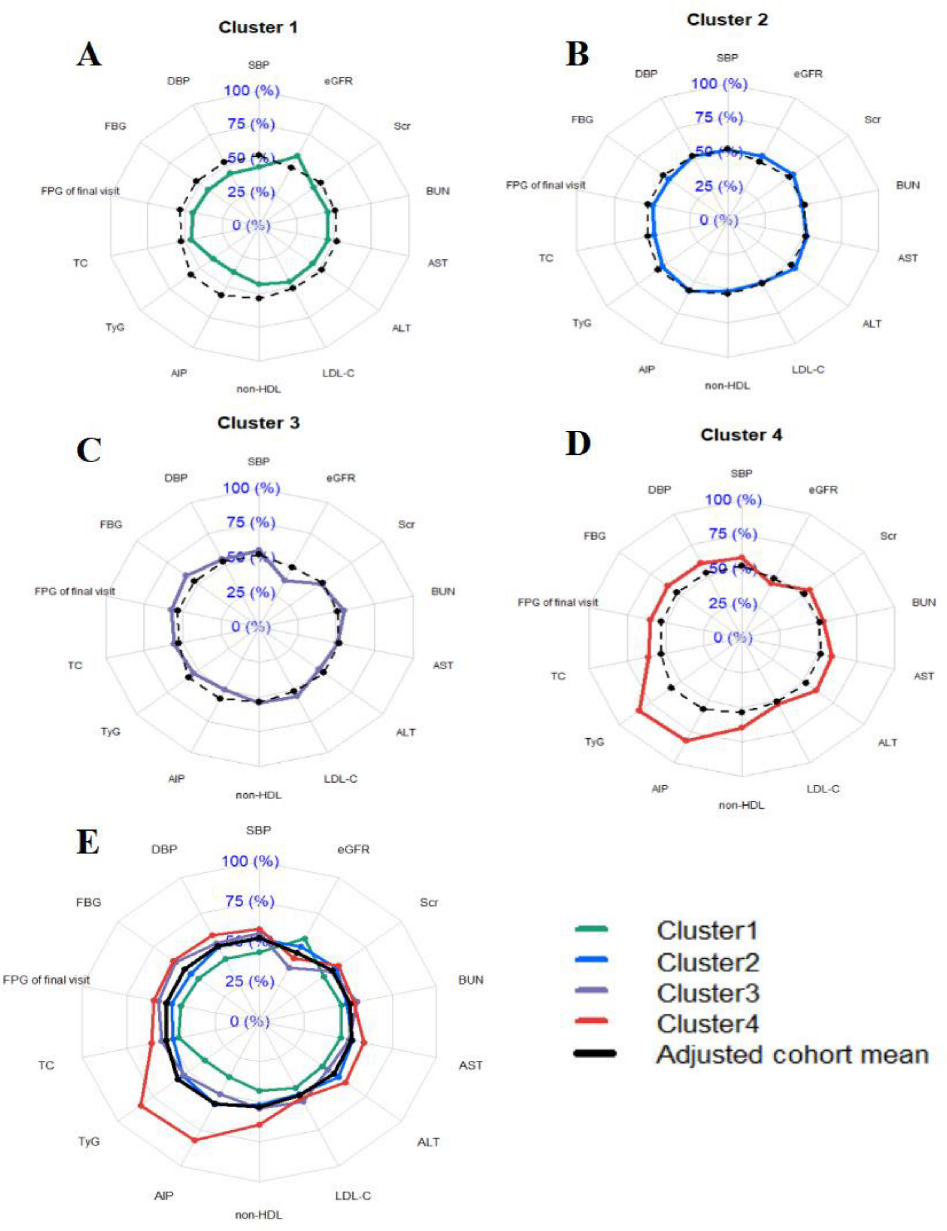
Profile of the four clusters in the cohort study. **(A–D)** Individual distributions of metabolic components in cluster 1, cluster 2, cluster 3 and cluster 4. **(E)** Combined distribution of metabolic components in clusters 1–4. Cluster 1: Metabolic health cluster; Cluster 2: Low HDL-C cluster; Cluster 3: Old age and mild metabolic disorder cluster; Cluster 4: Severe obesity and insulin resistance cluster. Radar plots were drawn for each cluster by using z-values which were calculated by adjusting the cluster mean for each variable to the cohort mean and SD for each variable. We then compared the radar plots visually and describe the particular characteristics of each cluster.

Table 2 provided the detailed overview of the distribution and clinical characteristics of the four clusters. The incidence of new-onset diabetes increased progressively from Cluster 1 to Cluster 4. Cluster 4 had the highest proportion of males and exhibited the highest levels of blood pressure (SBP and DBP), blood glucose (FBG at the final visit), lipid profiles (TC and non-HDL-C), and parameters reflecting IR, including AIP and TyG index. This cluster also showed the poorest liver function (highest ALT and AST levels) and suboptimal kidney function (elevated BUN, Scr, and reduced eGFR). Additionally, Cluster 4 had the highest rates of smoking, alcohol consumption, family history of diabetes and FBG_5_._6–6_._9_ (the proportion of people with FBG levels between 5.6 and 6.9 mmol/L). Similarly, Cluster 3 demonstrated relatively high levels of SBP, DBP, FBG at the final visit, TC, and non-HDL-C, along with elevated rates of smoking and alcohol consumption. This cluster also had the highest LDL-C and BUN levels but the lowest eGFR. The TyG index, AIP, Scr, ALT, and AST levels were moderate in this group. Cluster 2 exhibited lower levels of SBP, DBP, FBG at the final visit, TC, LDL-C, non-HDL-C, and BUN, along with lower rates of smoking and alcohol consumption. This cluster also had higher eGFR lever compared to the others. Cluster 1 had the lowest proportion of males and displayed the lowest levels of blood pressure (SBP and DBP), blood glucose (FBG at the final visit), and lipid profiles (TC, LDL-C, and non-HDL-C). It also had the lowest degree of IR (TyG and AIP) and the best liver and kidney function (lowest BUN, Scr, ALT, and AST, and highest eGFR). Additionally, this cluster had the lowest rates of smoking, alcohol consumption, family history of diabetes and FBG_5_._6–6_._9_. Detailed pairwise comparisons between clusters are presented in Table 2. Furthermore, using the adjusted cohort mean as a reference, radar charts (Figure 3) were generated to visually compare the clusters. These charts highlight that Cluster 4 exhibited significant metabolic disturbances, while Cluster 1 demonstrated optimal metabolic health. The characteristics of the study participants clustered by hierarchical clustering were grossly similar to those clustered by k-means clustering (Supplementary Table S3).

**TABLE 2:** Baseline clinical characteristics of the four clusters.

Variables	Cluster 1 (*n* = 4,784)	Cluster 2 (*n* = 3,146)	Cluster 3 (*n* = 3,781)	Cluster 4 (*n* = 896)	*P*-value
Age (years)	35.22 ± 6.60	37.25 ± 6.64^a^	56.41 ± 8.48^ab^	46.22 ± 9.48^abc^	< 0.001
Gender n, (%)					< 0.001
Male	2,323 (48.6)	3,203 (84.7)^a^	2,119 (67.4)^ab^	811 (90.5)^abc^	
Female	2,461 (51.4)	578 (15.3)	1,027 (32.6)	85 (9.5)	
Height (cm)	166.48 ± 8.15	170.39 ± 7.56^a^	165.99 ± 8.11^b^	170 ± 7.00^ac^	< 0.001
Weight (kg)	58.06 ± 8.34	75.19 ± 10.17^a^	66.74 ± 9.74^ab^	76.88 ± 10.47^abc^	< 0.001
BMI (kg/m^2^)	20.87 ± 1.96	25.84 ± 2.62^a^	24.15 ± 2.59^ab^	26.56 ± 2.93^abc^	< 0.001
SBP (mmHg)	112.93 ± 13.18	120.84 ± 13.79^a^	123.93 ± 16.58^ab^	126.32 ± 15.25^abc^	< 0.001
DBP (mmHg)	70.68 ± 9.18	76.09 ± 10.04^a^	77.53 ± 10.37^ab^	80.95 ± 10.50^abc^	< 0.001
FBG (mmol/L)	4.75 ± 0.54	4.93 ± 0.55^a^	5.35 ± 0.58^ab^	5.39 ± 0.61^ab^	< 0.001
FBG_5.6–6.9__*mmol/L*_ n, (%)	229 (4.8)	378 (10.0)^a^	1026 (32.6)^ab^	318 (35.5)^ab^	< 0.001
FBG of final visit (mmol/L)	4.99 ± 0.41	5.17 ± 0.55^a^	5.45 ± 0.70^ab^	5.55 ± 0.88^ab^	< 0.001
TC(mmol/L)	4.53 ± 0.80	4.69 ± 0.85^a^	5.04 ± 0.86^ab^	5.36 ± 1.02^abc^	< 0.001
TG(mmol/L)	0.89 ± 0.42	1.55 ± 0.65^a^	1.39 ± 0.60^ab^	4.29 ± 1.93^abc^	< 0.001
HDL-C (mmol/L)	1.48 ± 0.30	1.16 ± 0.23^a^	1.42 ± 0.27^ab^	1.19 ± 0.28^ac^	< 0.001
LDL-C (mmol/L)	2.56 ± 0.62	2.75 ± 0.68^a^	2.94 ± 0.68^ab^	2.84 ± 0.81^abc^	< 0.001
Non-HDL-C (mmol/L)	3.05 ± 0.74	3.53 ± 0.83^a^	3.62 ± 0.81^ab^	4.17 ± 0.98^abc^	< 0.001
TyG	8.04 ± 0.44	8.62 ± 0.45^a^	8.59 ± 0.47^ab^	9.75 ± 0.33^abc^	< 0.001
AIP	-0.25 ± 0.21	0.09 ± 0.21^a^	-0.04 ± 0.23^ab^	4.72 ± 1.11^abc^	< 0.001
BUN (mmol/L)	4.49 ± 1.12	4.72 ± 1.11^a^	5.12 ± 1.24^ab^	4.99 ± 1.16^ab^	< 0.001
Scr (umol/L)	69.24 ± 15.11	77.61 ± 13.92^a^	73.85 ± 15.20^ab^	78.86 ± 14.12^ac^	< 0.001
eGFR (ml/min/1.73 m^2^)	109.01 ± 12.19	104.59 ± 12.23^a^	92.23 ± 12.33^ab^	97.69 ± 13.06^abc^	< 0.001
ALT (U/L)	18.75 ± 16.83	33.62 ± 26.21^a^	23.57 ± 17.69^ab^	40.29 ± 25.85^abc^	< 0.001
AST (U/L)	22.39 ± 9.63	26.65 ± 11.26^a^	25.67 ± 10.04^a^	30.85 ± 11.65^abc^	< 0.001
Smoking status n, (%)					< 0.001
Current smoker	470 (9.8)	868 (23.0)^a^	843 (26.8)^ab^	343 (38.3)^abc^	
Ever smoker	153 (3.2)	232 (6.1)	122 (3.9)	56 (6.3)	
Never smoker	4161 (87.0)	2681 (70.9)	2181 (69.3)	497 (55.5)	
Drinking status n, (%)					< 0.001
Current drinker	60 (1.3)	109 (2.9)^a^	146 (4.6)^ab^	62 (6.9)^abc^	
Ever drinker	710 (14.8)	995 (26.3)	595 (18.9)	241 (26.9)	
Never drinker	4014 (83.9)	2677 (70.8)	2405 (76.4)	593 (66.2)	
Family histroy of diabetes n, (%)					< 0.001
Yes	254 (5.3)	276 (7.3)^a^	182 (5.8)	66 (7.4)^a^	
No	4530 (94.7)	3505 (92.7)	2964 (94.2)	830 (92.6)	
New-onset DM n, (%)	5 (0.1)	37 (1.0)^a^	147 (4.7)^ab^	62 (6.2)^abc^	< 0.001

Continuous variables are presented as the mean ± SD. All categorical variables were represented by numbers or proportions. Group comparisons of continuous variables are performed using ANOVA. When comparing pairwise, the least significant difference method was used. Group comparisons of categorical variables were conducted using the chi-square test. For multiple comparisons of proportions among multiple groups, Bonferroni correction is used to adjust the significance level. When *P* < 0.05, the difference is considered statistically significant. DM, diabetes mellitus; BMI, body mass index; SBP, systolic blood pressure; DBP, diastolic blood pressure; FBG, fasting blood glucose; TC, total cholesterol; TG, triglyceride; HDL-C, high-density lipoprotein cholesterol; LDL-C, low-density lipoprotein cholesterol; TyG, triglyceride-glucose index; AIP, atherogenic index of plasma; BUN, blood urea nitrogen; Scr, creatinine; eGFR, estimated glomerular filtration rate; ALT, alanine transferase; AST, aspartate transferase. ^a^*P* < 0.05 when cluster 1 is compared with cluster 2, cluster 3, and cluster 4. ^b^*P* < 0.05 when cluster 2 is compared with cluster 3 and cluster 4. ^c^*P* < 0.05 when cluster 3 is compared with cluster 4.

### 3.4 Association between new-onset diabetes and clusters

**FIGURE 4:**
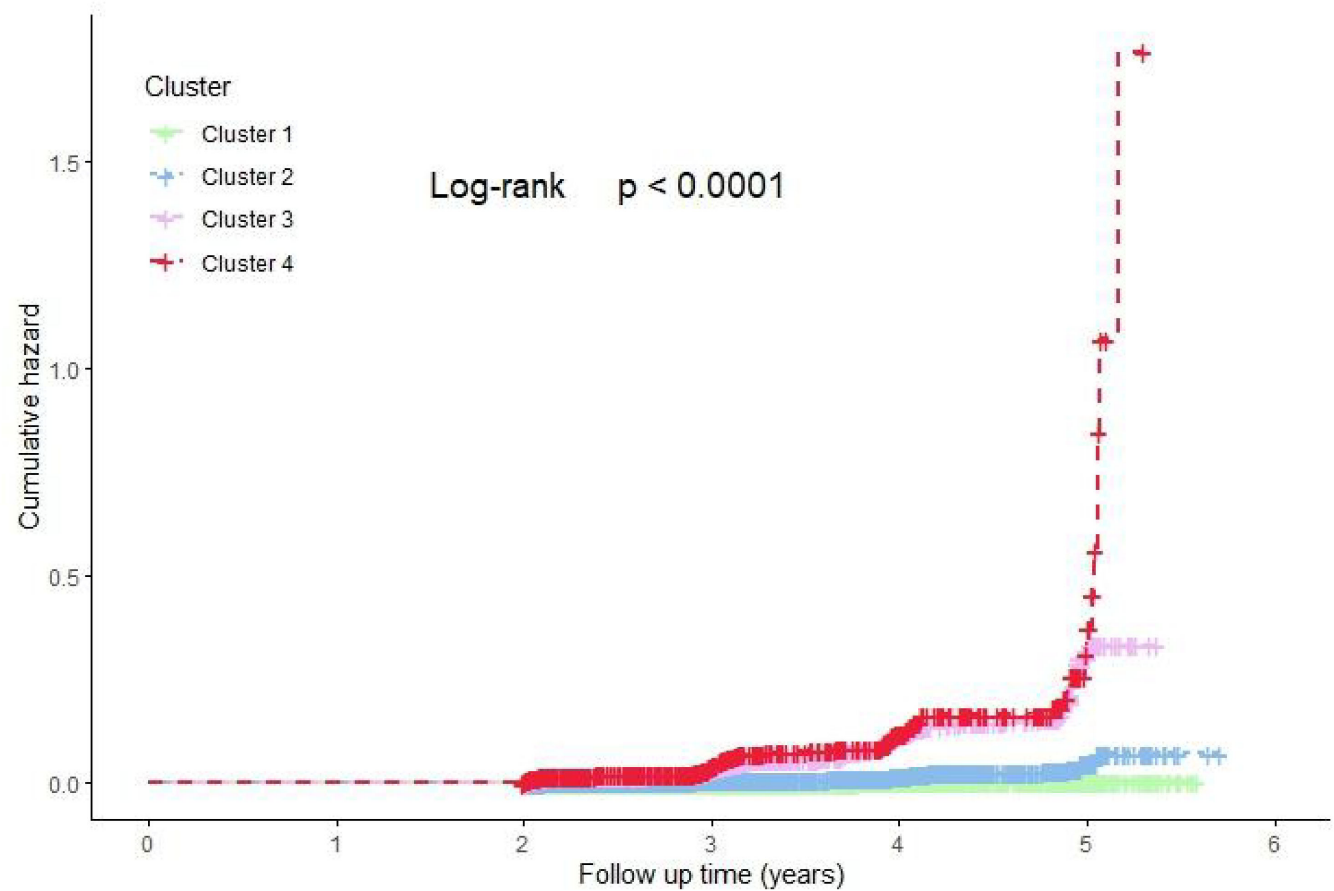
Kaplan-Meier estimated the cumulative hazard of new-onset DM risk among four clusters. Cluster 1: Metabolic health cluster; Cluster 2: Low HDL-C cluster; Cluster 3: Old age and mild metabolic disorder cluster; Cluster 4: Severe obesity and insulin resistance cluster.

The cumulative risk of diabetes incidence across the four clusters was analyzed using Kaplan-Meier (K-M) curves, as illustrated in Figure 4. The results revealed significant differences in the cumulative risk of diabetes among the four clusters over the follow-up period (Log-rank test, *P* < 0.0001). Clusters 3 and 4 exhibited a notably higher cumulative risk of diabetes incidence.

To further elucidate the association between different clusters and diabetes incidence, Cox proportional hazards regression models were employed. The detailed results were presented in Table 3. Compared to Cluster 1, Clusters 2, 3, and 4 showed significantly increased risks of diabetes incidence, consistent across all models with progressively adjusted covariates (unadjusted, Model 1, Model 2, and Model 3; *P* < 0.001). In pairwise comparisons, both Cluster 3 and Cluster 4 demonstrated significantly higher risks of diabetes incidence compared to Cluster 2, and these associations remained robust across all adjusted models (*P* < 0.001). However, no significant difference in diabetes risk was observed between Cluster 3 and Cluster 4 in any of the adjusted models (*P* > 0.05).

**TABLE 3:** Multiple Cox proportional hazard regression analysis for DM incidence according to clusters.

Cluster	Unadjusted		Model1		Model 2		Model 3	
	HR (95% CI)	*P*-value	HR (95% CI)	*P*-value	HR (95% CI)	*P*-value	HR (95% CI)	*P*-value
Cluster 1 vs. Cluster 2	9.64 (3.79–24.53)	< 0.001	8.86 (3.46–22.69)	< 0.001	8.52 (3.32–21.82)	< 0.001	7.15 (2.74–18.68)	< 0.001
Cluster 1 vs. Cluster 3	56.97 (23.36–138.95)	< 0.001	54.03 (22.09–132.13)	< 0.001	51.96 (21.23–127.20)	< 0.001	25.11 (9.61–65.61)	< 0.001
Cluster 1 vs. Cluster 4	70.23 (28.23–174.68)	< 0.001	63.59 (25.34–159.58)	< 0.001	58.54 (23.27–147.27)	< 0.001	32.04 (11.92–86.07)	< 0.001
Cluster 2 vs. Cluster 3	5.91 (4.12–8.48)	< 0.001	6.10 (4.24–8.77)	< 0.001	6.10 (4.23–8.80)	< 0.001	3.51 (2.19–5.63)	< 0.001
Cluster 2 vs. Cluster 4	7.28 (4.85–10.95)	< 0.001	7.18 (4.77–10.79)	< 0.001	6.88 (4.56–10.37)	< 0.001	4.48 (2.80–7.17)	< 0.001
Cluster 3 vs. Cluster 4	1.23 (0.92–1.66)	0.168	1.18 (0.87–1.59)	0.292	1.13 (0.83–1.53)	0.443	1.28 (0.86–1.90)	0.232

Unadjusted. Model 1: adjusted for gender. Model 2: adjusted for gender, smoking status, drinking status, and family histroy of diabetes. Model 3: adjusted for gender, SBP, DBP, TC, non-HDL, LDL, ALT, AST, BUN, Scr, eGFR, smoking status, drinking status, and family history of diabetes.

We have detailed the distribution of characteristic levels and the results of pairwise comparison tests between clusters (Clusters 1, 2, 3, and 4) within both male and female subgroups. These results align well with the metabolic level comparisons in the overall population (Supplementary Figures S2–S5; Supplementary Tables S5, S6). Similarly, we analyzed the risk of diabetes incidence across clusters within male and female subgroups separately (Supplementary Table S7). The findings demonstrated that, in fully adjusted models, Clusters 3 and 4 had significantly higher risks of diabetes incidence compared to Clusters 1 and 2 (*P* < 0.05). However, no significant difference in diabetes risk was observed between Clusters 3 and 4 (*P* > 0.05), aligning with the results from the overall population.

## 4 Discussion

This study conducted clustering analysis using five easily accessible clinical indicators (age, BMI, FBG, TG, and HDL-C) and identified four distinct clusters with significant characteristic differences within the population. The characteristics of the study participants derived from k-means clustering were essentially consistent with those obtained by hierarchical clustering. These clusters were labeled as MHC (Cluster 1), LHC (Cluster 2), OMDC (Cluster 3), and SOIRC (Cluster 4). At the end of the follow-up period, Cluster 4 exhibited the highest incidence of diabetes, followed by Cluster 3, Cluster 2, and Cluster 1. Furthermore, in multiple models adjusted for covariates, the diabetes incidence rates in Cluster 3 and Cluster 4 were significantly higher than those in Cluster 1 and Cluster 2, although no significant difference was observed between Cluster 3 and Cluster 4. These findings were consistently validated across different genders. The results suggest that clustering analysis can effectively reveal the heterogeneity in diabetes incidence among clusters with distinct metabolic profiles, highlighting the need for early intervention in high-risk populations characterized by aging, obesity, and IR.

In this study, Cluster 4 had the highest incidence of diabetes and exhibited the worst metabolic profile, characterized by hypertension (elevated SBP and DBP), dysregulated glucose and lipid metabolism (high FBG, TC, LDL-C, and Non-HDL-C, and low HDL-C), and impaired liver and kidney function (elevated ALT, AST, BUN, Scr, and reduced eGFR). This may be related to the cluster’s IR (elevated TyG (23) and AIP (24), which have been identified as surrogate markers of IR) and obesity status (high BMI). In multiple studies focusing on newly diagnosed patients with T2DM, IR- and obesity-related subgroups have indeed demonstrated severe metabolic disturbances and a high incidence of complications (7, 9, 25, 26).

Dyslipidemia is one of the common complications of T2DM, with a prevalence as high as 72–85% (27). Under conditions of IR, elevated levels of free fatty acids (FFA) in the circulation lead to increased hepatic synthesis of very low-density lipoprotein (VLDL). Additionally, reduced activity of lipoprotein lipase (LPL) contributes to decreased VLDL degradation, ultimately resulting in hypertriglyceridemia (28). Elevated TG activates cholesterol ester transfer protein (CETP), promoting the transfer of TG from triglyceride-rich lipoproteins (TRLs) to HDL-C and LDL-C. TG-rich HDL-C and its surface apolipoprotein AI (ApoAI) are rapidly cleared, while TG-rich LDL-C is transformed into sdLDL (29). Under the combined influence of these lipid abnormalities, individuals with T2DM are prone to vascular endothelial dysfunction, hypertension, atherosclerosis (AS), and cardiovascular diseases (CVD). Ultimately, approximately 70–80% of individuals die from cardiovascular and cerebrovascular diseases (30). Therefore, close attention to lipid profiles in Cluster 4 is essential in the early stages.

Obesity is another critical factor that cannot be overlooked in Cluster 4. On one hand, increased lipolysis in obese individuals leads to elevated FFA entering the liver and muscles. This can cause mitochondrial dysfunction, endoplasmic reticulum stress (ERS), or ectopic fat deposition, interfering with insulin signaling (e.g., insulin receptor substrate 1 (IRS-1) phosphorylation) and resulting in reduced glucose uptake and impaired glucose tolerance (31–33). On the other hand, the accumulation of visceral fat promotes the release of inflammatory cytokines such as tumor necrosis factor-α (TNF-α) and interleukin-6 (IL-6) from adipocytes. These cytokines can circulate through the bloodstream and affect organs like the liver and muscles, exacerbating adipose and systemic IR and creating the vicious cycle (34). These mechanisms have been well-documented as significant contributors to T2DM and impaired liver and kidney function (35, 36). Therefore, weight reduction is essential.

In this study, Cluster 3 had the oldest participants and exhibited a relatively poor metabolic profile. The risk of developing diabetes in this cluster was not significantly different from that in Cluster 4 but was higher than in Cluster 1 and Cluster 2. A study in Korea found that the oldest subgroup had the highest levels of C-reactive protein (CRP) (37), and the risk of T2DM in this subgroup was similar to that in the IR subgroup, which aligned with the findings of this study. Unfortunately, the database used in this study did not include inflammation-related indicators. Research has shown that aging is significantly associated with a persistent increase in systemic pro-inflammatory cytokine levels (38). Age-dependent accumulation of visceral fat can induce adipocyte hypertrophy and the formation of a hypoxic microenvironment, driving macrophages to polarize toward the M1 phenotype and secrete large amounts of inflammatory mediators (e.g., TNF-α and IL-6) (39, 40). These cytokines activate serine phosphorylation sites (e.g., Ser307) on IRS-1, hindering its normal binding to the insulin receptor and ultimately disrupting the PI3K-Akt signaling pathway (39, 40). Additionally, age-related hormonal remodeling significantly exacerbates metabolic imbalances. The progressive decline in the growth hormone (GH)/insulin-like growth factor-1 (IGF-1) axis leads to an annual loss of skeletal muscle mass by 1–2%. Reduced muscle glucose uptake capacity directly impairs systemic insulin sensitivity, contributing to the development of T2DM (41). These mechanisms have been validated in large-scale epidemiological studies (42, 43). Therefore, elderly patients may benefit from anti-inflammatory diets [e.g., the Mediterranean diet, which reduces CRP levels and improves insulin sensitivity (44)] and regular exercise [e.g., resistance training and aerobic exercise, which counteract muscle loss and IR (45)] to prevent T2DM.

Compared to other clusters, Cluster 1 and Cluster 2 exhibited lower incidences of diabetes. Among these, Cluster 1 displayed the healthiest metabolic profile, characterized by the lowest levels of blood pressure, blood glucose, lipid profiles, and IR, as well as optimal liver and kidney function. The low-risk features of Cluster 1 may stem from the synergistic effects of genetic factors and healthy behaviors, as evidenced by the lowest proportions of family history of diabetes, smoking, and alcohol consumption. Studies have confirmed that specific genetic traits and healthy lifestyles can enhance insulin sensitivity and strengthen metabolic protective effects (46, 47).

Notably, although Cluster 2 demonstrated relatively favorable metabolic characteristics (as shown in the radar chart) and a significant age advantage (mean age of 37.25 years), its incidence and risk of new-onset diabetes were still significantly higher than those of Cluster 1. This phenomenon suggests that traditional metabolic indicators may not fully capture early pathological changes, and unaccounted environmental exposure factors may play a critical role in young and middle-aged populations. For instance, long-term consumption of highly processed foods (48), chronic stress exposure (49), sleep deprivation (50), and environmental endocrine disruptors (51) can exacerbate metabolic disturbances through multiple mechanisms. In the future, incorporating non-traditional risk factors such as dietary patterns, stress load, circadian rhythms, and environmental toxins could provide a multidimensional explanation for diabetes risk. These factors may initiate β-cell exhaustion during the metabolic compensation phase (e.g., the compensatory hyperinsulinemia stage), ultimately leading to overt T2DM.

In this study, we have detailed the distribution of characteristic levels and the results of pairwise comparison tests between clusters (Clusters 1, 2, 3, and 4) within both male and female subgroups. These results align well with the metabolic level comparisons in the overall population. This consistency supports the robustness of our primary analysis while providing nuanced gender-specific insights through subgroup comparisons.

The strengths of this study lie in the fact that age, BMI, and glucose-lipid metabolic indicators are easily obtainable during routine health check-ups in the general healthy population. By employing clustering analysis based on these routine clinical indicators, we effectively identified high-risk individuals for diabetes in the Chinese population, providing a scientific basis for targeted early intervention and reducing the additional healthcare burden caused by disease progression. However, this study also has several limitations. First, the sample was derived exclusively from the Chinese adult population, necessitating caution when extrapolating the findings to other populations. Moreover, only baseline data were used, and clustering indicators at multiple time points were not recorded. Future studies should incorporate longitudinal designs with longer follow-up periods to explore the association between metabolic trajectories and T2DM. Third, due to sample size constraints, we did not perform sex-stratified clustering, although subgroup analyses demonstrated consistent cluster characteristics across genders. Future studies with larger cohorts are needed to validate. Fourth, the absence of HbA1c data should be noted as a limitation. As a well-established marker of long-term glycemic control, HbA1c could have offered supplementary perspectives on glucose homeostasis. Subsequent investigations incorporating HbA1c measurements may further validate our clustering results. Finally, the clustering indicators (Age, BMI, FBG, TG, HDL-C) selected in this study may not be comprehensive. In subsequent research, multi-omics data (e.g., gut microbiota, epigenetic markers) could be included to provide a more detailed understanding of the heterogeneity of T2DM.

## 5 Conclusion

Clustering analysis, based on simple and easily measurable clinical indicators, can effectively identify individuals at high risk of developing diabetes. Aging, obesity, and IR are significant risk factors for diabetes onset. Early identification of such populations and the implementation of targeted interventions (such as improving glucose and lipid metabolism, enhancing insulin sensitivity, and controlling body weight) may help delay the progression of T2DM and reduce the burden of complications. Future studies should incorporate multidimensional data to further validate clusters characteristics, thereby providing the theoretical foundation for precision medicine.

## Data Availability

The datasets presented in this study can be found in online repositories. The names of the repository/repositories and accession number(s) can be found in the article/Supplementary material.

## Footnotes

1
https://datadryad.org/dataset/doi:10.5061/dryad.ft8750v
