# Supplementary material for: Data-driven cluster analysis on the association of aging, obesity and insulin resistance with new-onset diabetes in Chinese adults: a multicenter retrospective cohort study
Source: Front Med (Lausanne). 2025 Jul 30;12:1640017. doi: 10.3389/fmed.2025.1640017 (PMC12343622; doi:10.3389/fmed.2025.1640017)
Supplement: Supplementary file 1 [file Supplementary_file_1.pdf]

Supplements

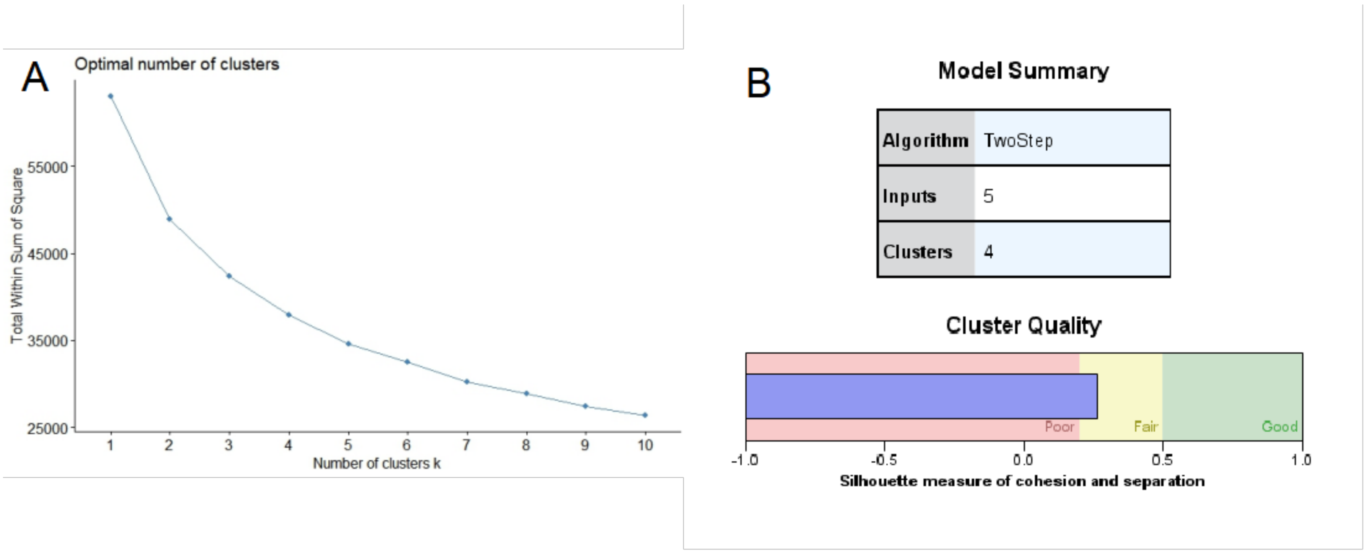

FIGURE S1 Determination of k in k-means clustering models. A. Elbow plot; B.Two-step cluster.

Note: Based on five input variables (Age, BMI, FBG, TG, and HDL-C), the algorithm generated four distinct clusters, hinting the optimal number of clusters is 4.

TABLE S1. Average silhouette width for different numbers k-means of clusters

Number of clusters	Average silhouette width
2	0.255
3	0.230
4	**0.280**
5	0.226
6	0.160
7	0.164
8	0.166
9	0.152
10	0.155
11	0.152
12	0.157
13	0.152
14	0.154
15	0.151

TABLE S2. Average silhouette width for different numbers of hierarchical clusters

Number of clusters	Average silhouette width
2	0.262
3	0.267
4	**0.276**
5	0.215
6	0.157
7	0.165
8	0.167
9	0.148
10	0.152
11	0.155
12	0.158
13	0.152
14	0.149
15	0.151

TABLE S3. Baseline characteristics of study participants by hierarchical clustering.

**Variables**	**Cluster 1 (n=4947)**	**Cluster 2 (n=3766)**	**Cluster 3 (n=3180)**	**Cluster 4 (n=714)**	***P* value**
Age (years)	36.52±7.63	40.75±7.38[a]	58.26±8.64[ab]	43.27±11.45[ac]	<0.001
Male n, (%)	2231 (45.1)	3013 (80.0)[a]	2162 (70.3)[ab]	597 (83.6)[ac]	<0.001
Height (cm)	165.83±8.14	168.15±8.05[a]	169.18±7.78[ab]	169.26±8.23[ab]	<0.001
Weight (kg)	54.93±6.69	76.43±7.07[a]	66.13±8.06[ab]	78.65±10.11[abc]	<0.001
BMI (kg/m2)	19.92±1.31	26.43±0.97[a]	23.54±1.40[ab]	28.87±1.94[abc]	<0.001
SBP (mmHg)	112.82±13.73	119.08±14.78[a]	123.76±14.56[ab]	129.88±15.53[abc]	<0.001
DBP (mmHg)	70.71±9.17	74.61±9.95[a]	78.14±10.37[ab]	81.75±10.88[abc]	<0.001
FBG (mmol/L)	4.86±0.55	5.02±0.59[a]	5.15±0.61[ab]	5.22±0.64[abc]	<0.001
FBG5.6-6.9 (mmol/L) n, (%)	247 (5.0)	580 (15.4)[a]	894 (28.1)[ab]	256 (35.8)[abc]	<0.001
FBG of final visit (mmol/L)	5.01±0.45	5.21±0.57[a]	5.32±0.68[ab]	5.50 ±0.93[abc]	<0.001
TC (mmol/L)	4.55±0.83	4.78±0.85[a]	4.96±0.92[ab]	5.04±0.93[ab]	<0.001
TG (mmol/L)	0.97±0.56	1.45±1.06[a]	1.92±1.33[ab]	4.17±1.35[abc]	<0.001
HDL-C (mmol/L)	1.46±0.29	1.20±0.30[a]	1.27±0.27[ab]	1.24±0.27[ab]	<0.001
LDL-C (mmol/L)	2.57±0.63	2.76±0.66[a]	2.97±0.72[ab]	2.92±0.72[ab]	<0.001
Non-HDL-C (mmol/L)	3.09±0.76	3.44±0.89[a]	3.70±0.83[ab]	3.97±0.91[abc]	<0.001
TyG	8.10±0.49	8.49±0.59[a]	8.81±0.58[ab]	8.95±0.59[abc]	<0.001
AIP	-0.22±0.24	0.12±0.28[a]	-0.03±0.27[ab]	4.19±0.27[abc]	<0.001
BUN (mmol/L)	4.52±1.16	4.79±1.15[a]	4.93±1.19[ab]	4.91±1.19[ab]	<0.001
SCr (umol/L)	68.29±14.92	74.41±14.73[a]	77.84±14.49[ab]	79.49±14.55[ab]	<0.001
eGFR (ml/min/1.73m2)	107.13±13.45	101.89±13.57[a]	99.02±13.96[ab]	100.14±14.27[ab]	<0.001
ALT (U/L)	17.81±17.42	24.86±18.98[a]	33.09±23.78[ab]	45.57±33.50[abc]	<0.001
AST (U/L)	22.48±9.80	24.60±9.62[a]	27.44±10.88[ab]	32.17±15.05[abc]	<0.001
Smoking status n, (%)					<0.001
current smoker	613 (12.4)	746 (19.8)[a]	875 (27.5)[ab]	205 (28.7)[ab]	
ever smoker	129 (2.6)	173 (4.6)	184 (5.8)	51 (7.1)	
never smoker	4205 (85.0)	2847 (75.6)	2121 (66.7)	458 (64.2)	
Drinking status n, (%)					<0.001
current drinker	74 (1.5)	94 (2.5)[a]	162 (5.1)[ab]	49 (6.9)[abc]	
ever drinker	619 (12.5)	829 (22.0)	801 (25.2)	184 (25.8)	
never drinker	4254 (86.0)	2843 (75.5)	2217 (69.7)	481 (67.3)	
Family histroy of diabetes n, (%)					<0.001
Yes	247 (5.0)	252 (6.7)[a]	200 (6.3)[a]	56 (7.8)[a]	
No	4700 (95.0)	3514 (93.3)	2980 (93.7)	658 (92.2)	
New-onset DM n, (%)	15 (0.3)	68 (1.8)[a]	102 (3.2)[ab]	44 (6.2)[abc]	<0.001

Continuous variables are presented as the mean ± SD. All categorical variables were represented by numbers or proportions. Group comparisons of continuous variables are performed using ANOVA. When comparing pairwise, the least significant difference method was used. Group comparisons of categorical variables were conducted using the chi-square test. For multiple comparisons of proportions among multiple groups, Bonferroni correction is used to adjust the significance level. When $P<0.05$, the difference is considered statistically significant.

Abbreviations: DM, diabetes mellitus; BMI, body mass index; SBP, systolic blood pressure; DBP, diastolic blood pressure; FBG, fasting

blood glucose; TC, total cholesterol; TG, triglyceride; HDL-C, high-density lipoprotein cholesterol; LDL-C, low-density lipoprotein cholesterol; TyG, triglyceride-glucose inde；AIP, atherogenic index of plasma; BUN, blood urea nitrogen; Scr, creatinine; eGFR, estimated glomerular filtration rate; ALT, alanine transferase; AST, aspartate transferase.

[a] indicates $P<0.05$ when cluster 1 is compared with cluster 2, cluster 3, and cluster 4

[b] indicates $P<0.05$ when cluster 2 is compared with cluster 3 and cluster 4

[c] indicates $P<0.05$ when cluster 3 is compared with cluster 4

TABLE S4. Cluster center coordinates.

Cluster	Age	BMI	FBG	TG	HDL-C
Cluster 1	-0.5827729	-0.8244466	-0.4041890	-0.5056275	0.4330349
Cluster 2	-0.4056082	0.6857378	-0.1103921	0.0840325	-0.6033898
Cluster 3	1.2661468	0.1722173	0.5689239	-0.0555297	0.2181925
Cluster 4	0.3775587	0.9035515	0.6263371	2.5400575	-0.5319817

BMI, body mass index; FBG, fasting blood glucose; TG, triglyceride; HDL-C, high-density lipoprotein cholesterol.
Cluster 1: Metabolic health cluster; Cluster 2: Low HDL-C cluster; Cluster 3: Old age and mild metabolic disorder cluster; Cluster 4: Severe obesity and insulin resistance cluster.

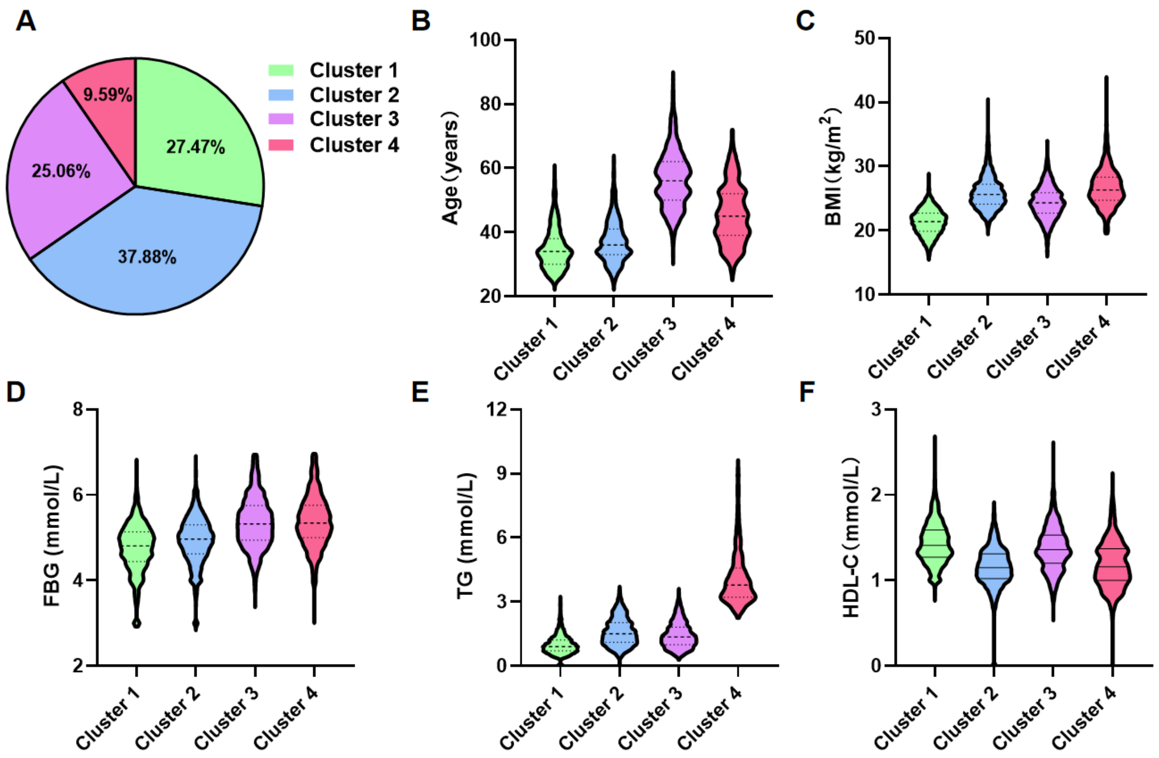

FIGURE S2 Distribution and clinical features of clusters in men. (A) Proportional distribution of male participants. (B-F) Characteristics of each cluster regarding age, BMI, FBG, TG, and HDL-C in male participants. Cluster 1: Metabolic health cluster; Cluster 2: Low HDL-C cluster; Cluster 3: Old age and mild metabolic disorder cluster; Cluster 4: Severe obesity and insulin resistance cluster.

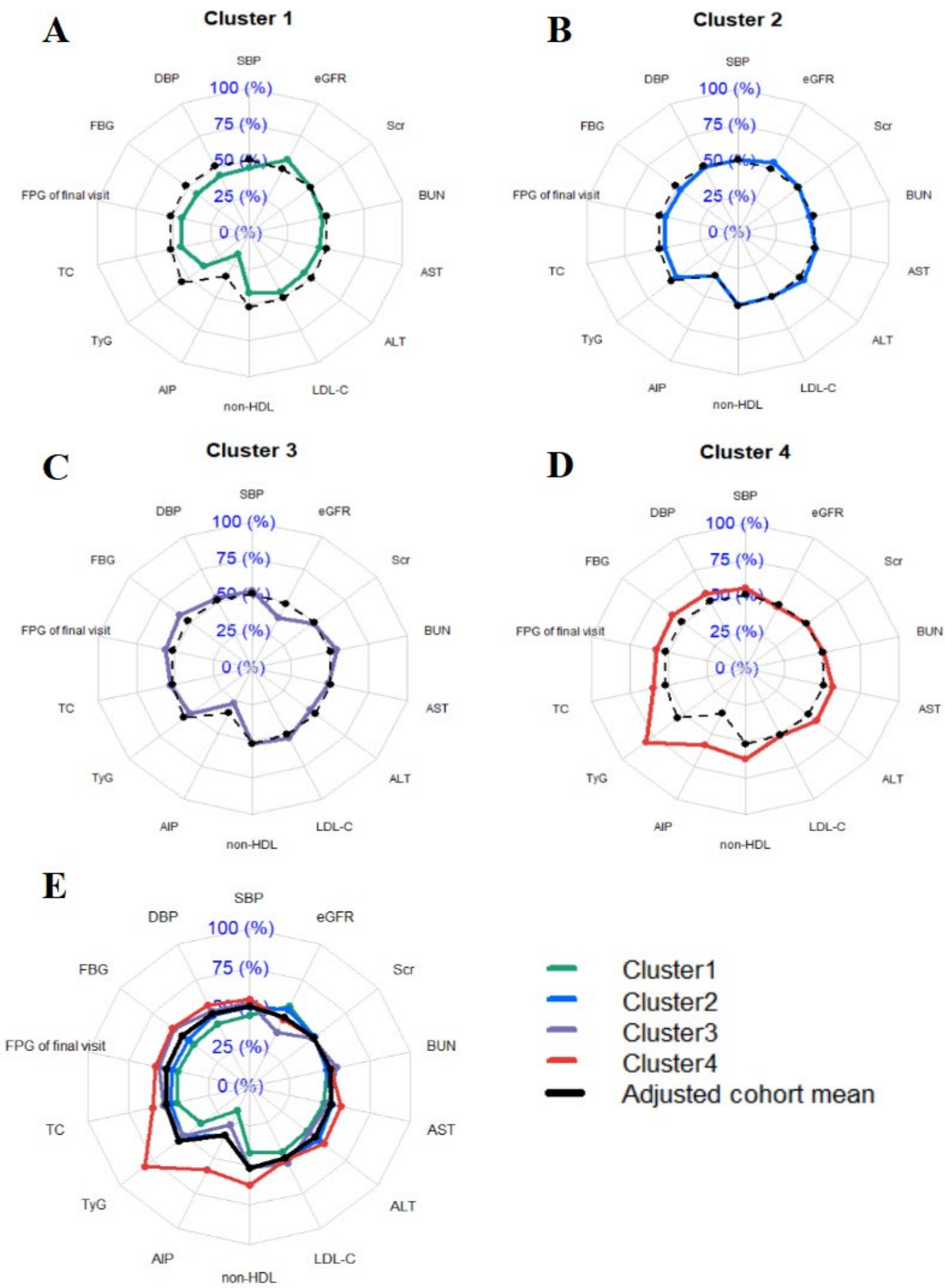

FIGURE S3 Profile of the four clusters in the cohort study for men. (A-D) Individual distributions of metabolic components in cluster 1, cluster 2, cluster 3 and cluster 4 among male participants. (E) Combined distribution of metabolic components in clusters 1-4 among male participants among male participants. Cluster 1: Metabolic health cluster; Cluster 2: Low HDL-C cluster; Cluster 3: Old age and mild metabolic disorder cluster; Cluster 4: Severe obesity and insulin resistance cluster. Radar plots were drawn for each cluster by using z-values which were calculated by adjusting the cluster mean for each variable to the cohort mean and SD for each variable. We then compared the radar plots visually.

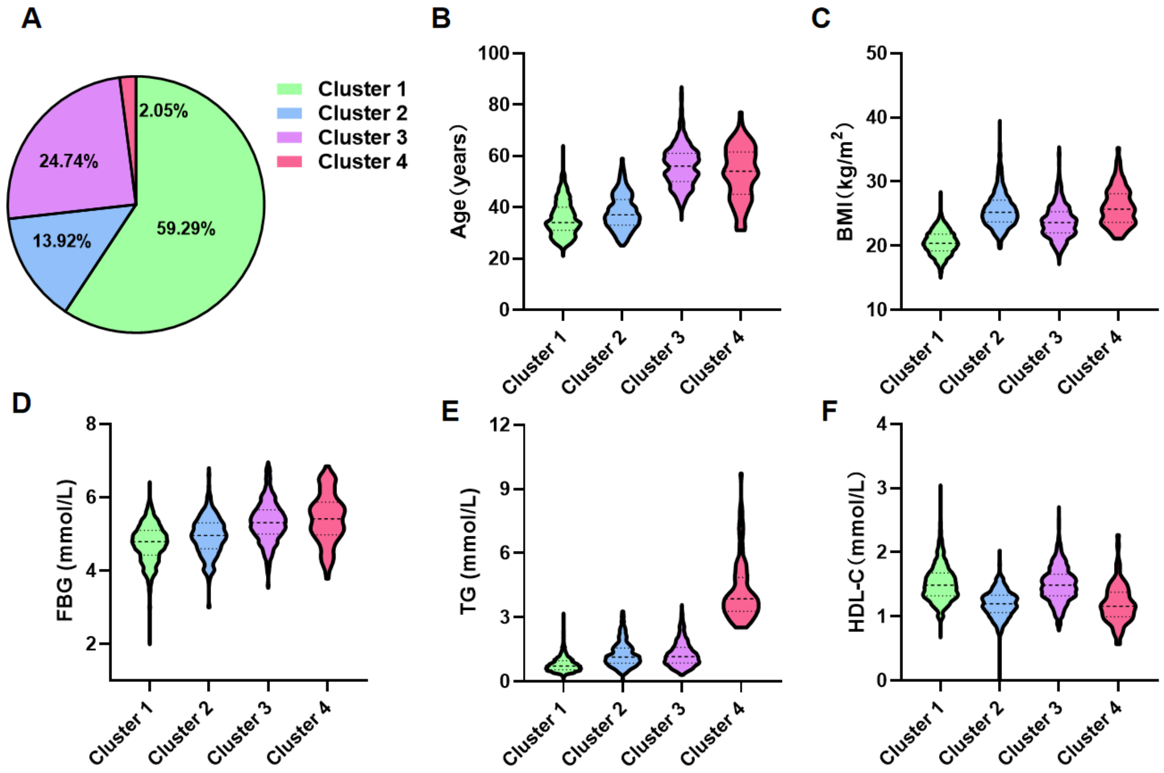

FIGURE S4 Distribution and clinical features of clusters in women. (A) Proportional distribution of female participants. (B-F) Characteristics of each cluster regarding age, BMI, FBG, TG, and HDL-C in female participants. Cluster 1: Metabolic health cluster; Cluster 2: Low HDL-C cluster; Cluster 3: Old age and mild metabolic disorder cluster; Cluster 4: Severe obesity and insulin resistance cluster.

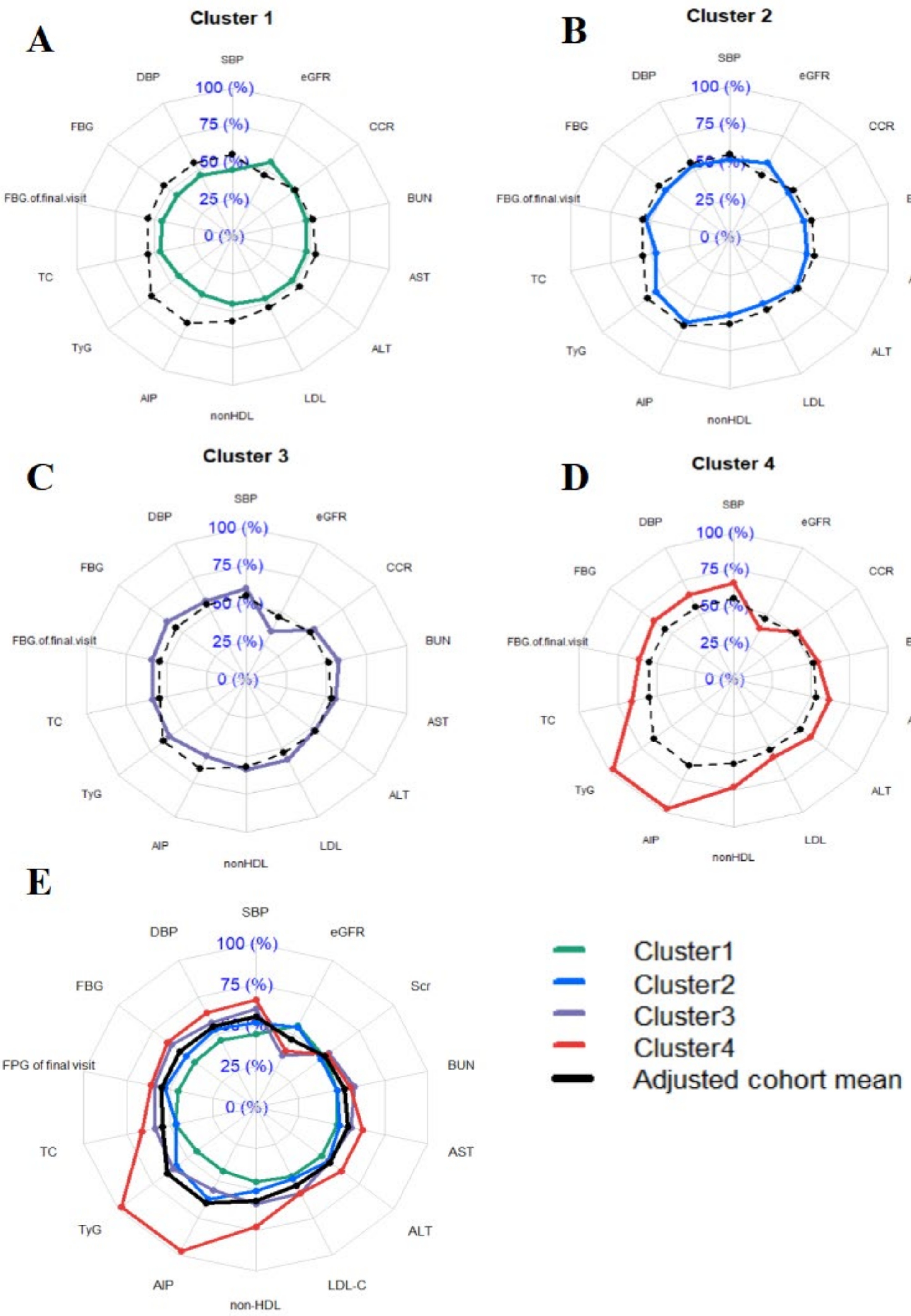

FIGURE S5 Profile of the four clusters in the cohort study for women. (A-D) Individual distributions of metabolic components in cluster 1, cluster 2, cluster 3 and cluster 4 among female participants. (E) Combined distribution of metabolic components in clusters 1-4 among female participants. Cluster 1: Metabolic health cluster; Cluster 2: Low HDL-C cluster; Cluster 3: Old age and mild metabolic disorder cluster; Cluster 4: Severe obesity and insulin resistance cluster. Radar plots were drawn for each cluster by using z-values which were calculated by adjusting the cluster mean for each variable to the cohort mean and SD for each variable. We then compared the radar plots visually.

TABLE S5. Metabolic characteristics of the four clusters in male subgroup

**Variables**	**Overall (n=8456)**	**Cluster 1 (n=2323)**	**Cluster 2 (n=3203)**	**Cluster 3 (n=2119)**	**Cluster 4 (n=811)**	***P* value**
Age (years)	42.12±11.50	34.77±6.37	37.06±6.58^{a}	56.52±8.76^{ab}	45.50±9.08^{abc}	<0.001
Height (cm)	171.61±6.16	172.48±5.90	172.30±6.12	169.77±6.18^{ab}	171.26±5.88^{abc}	<0.001
Weight (kg)	71.70±10.49	63.48±7.18	76.98±9.44^{a}	70.24±8.74^{ab}	78.11±9.92^{abc}	<0.001
BMI (kg/m2)	24.31±3.16	21.29±1.98	25.89±2.59^{a}	24.34±2.54^{ab}	26.61±2.92^{abc}	<0.001
SBP (mmHg)	121.61±14.52	117.21±12.77	121.85±13.47^{a}	124.24±16.29^{ab}	126.16±14.96^{abc}	<0.001
DBP (mmHg)	76.48±10.10	72.87±8.90	76.68±9.87^{a}	78.33±10.24^{ab}	81.06±10.47^{abc}	<0.001
FBG (mmol/L)	5.04±0.62	4.76±0.57	4.93±0.55^{a}	5.36±0.59^{ab}	5.38±0.60^{ab}	<0.001
FBG5.6-6.9 (mmol/L) n, (%)	1450 (17.1)	129 (5.5)	319 (10.0)^{a}	719 (33.9)^{ab}	283 (34.9)^{ab}	<0.001
FBG of final visit (mmol/L)	5.24±0.65	5.02±0.42	5.16±0.56^{a}	5.48±0.73^{ab}	5.56±0.89^{ab}	<0.001
TC (mmol/L)	4.80±0.89	4.55±0.82	4.74±0.85^{a}	4.98±0.85^{ab}	5.34±0.98^{abc}	<0.001
TG (mmol/L)	1.65±1.22	0.99±0.44	1.60±0.65^{a}	1.44±0.60^{ab}	4.29±1.98^{abc}	<0.001
HDL-C (mmol/L)	1.29±0.30	1.44±0.31	1.16±0.24^{a}	1.37±0.26^{ab}	1.18±0.27^{ac}	<0.001
LDL-C (mmol/L)	2.77±0.69	2.60±0.63	2.79±0.68^{a}	2.92±0.67^{ab}	2.83±0.79^{ac}	<0.001
Non-HDL-C (mmol/L)	3.51±0.88	3.10±0.78	3.57±0.83^{a}	3.61±0.81^{a}	4.15±0.96^{abc}	<0.001
TyG	8.62±0.61	8.16±0.43	8.66±0.44^{a}	8.63±0.46^{a}	9.75±0.33^{abc}	<0.001
AIP	0.04±0.29	-0.19±0.20	0.11±0.21^{a}	-0.01±0.22^{ab}	0.54±0.17^{abc}	<0.001
BUN (mmol/L)	4.95±1.16	4.82±1.11	4.81±1.11	5.25±1.22^{ab}	5.02±1.14^{abc}	<0.001
SCr (umol/L)	81.01±11.60	81.11±10.99	81.36±11.11	80.43±12.52^{b}	80.88±12.58	<0.001
eGFR (ml/min/1.73m2)	100.10±13.31	105.25±11.82	103.29±11.96^{a}	90.73±11.92^{ab}	97.64±12.61^{abc}	<0.001
ALT (U/L)	30.20±23.62	22.67±17.26	36.12±26.93^{a}	25.19±18.73^{ab}	41.48±26.30^{abc}	<0.001
AST (U/L)	26.51±11.38	23.97±11.22	27.56±11.50^{a}	25.95±10.44^{ab}	31.12±11.79^{abc}	<0.001
New-onset DM n, (%)	203 (2.4)	3 (0.1)	33 (1.0)	111 (5.2)	56 (6.9)	<0.001

Abbreviations: BMI, body mass index; SBP, systolic blood pressure; DBP, diastolic blood pressure; FBG, fasting blood glucose; TC, total cholesterol; TG, triglyceride; HDL-C, high-density lipoprotein cholesterol; LDL-C, low-density lipoprotein cholesterol; TyG, triglyceride-glucose inde；AIP, atherogenic index of plasma; BUN, blood urea nitrogen; Scr, creatinine; eGFR, estimated glomerular filtration rate; ALT, alanine transferase; AST, aspartate transferase; DM, diabetes mellitus.

TABLE S6. Metabolic characteristics of the four clusters in female subgroup

Variables	Overall (n=4151)	Cluster 1 (n=2461)	Cluster 2 (n=578)	Cluster 3 (n=1027)	Cluster 4 (n=85)	*P* value
Age (years)	41.46±11.37	35.64±6.77	38.26±6.83[a]	56.16±7.87[ab]	53.16±10.45[abc]	<0.001
Height (cm)	159.84±5.67	160.78±5.44	159.81±5.86[a]	158.20±5.70[ab]	157.89±4.93[ab]	<0.001
Weight (kg)	56.59±8.17	52.98±5.80	65.45±8.25[a]	59.52±7.45[ab]	65.19±8.25[ac]	<0.001
BMI (kg/m2)	22.12±3.05	20.48±1.85	25.61±2.77[a]	23.77±2.63[ab]	26.12±3.00[ac]	<0.001
SBP (mmHg)	113.80±15.43	108.95±12.30	115.35±14.20[a]	123.29±17.17[ab]	128.24±17.71[abc]	<0.001
DBP (mmHg)	71.28±10.19	68.65±9.00	73.03±10.47[a]	75.86±10.45[ab]	79.94±10.90[abc]	<0.001
FBG (mmol/L)	4.94±0.57	4.75±0.50	4.95±0.51[a]	5.33±0.55[ab]	5.42±0.68[ab]	<0.001
FBG5.6-6.9 (mmol/L) n, (%)	501 (12.1)	100 (4.1)	59 (10.2)[a]	307 (29.9)[ab]	35 (41.2)[abc]	<0.001
FBG of final visit (mmol/L)	5.10±0.51	4.95±0.39	5.18±0.48[a]	5.38±0.61[ab]	5.44±0.77[ab]	<0.001
TC (mmol/L)	4.70±0.87	4.52±0.78	4.49±0.79	5.15±0.87[ab]	5.54±1.30[ab]	<0.001
TG (mmol/L)	1.06±0.71	0.80±0.36	1.28±0.59[a]	1.28±0.58[a]	4.21±1.38[abc]	<0.001
HDL-C (mmol/L)	1.47±0.29	1.52±0.28	1.20±0.20[a]	1.50±0.28[b]	1.20±0.30[ac]	<0.001
LDL-C (mmol/L)	2.66±0.67	2.53±0.60	2.59±0.64[a]	2.99±0.70[ab]	2.98±0.96[ab]	<0.001
Non-HDL-C (mmol/L)	3.23±0.81	3.00±0.69	3.28±0.76[a]	3.64±0.81[ab]	4.34±1.17[abc]	<0.001
TyG	8.17±0.56	7.93±0.42	8.42±0.46[a]	8.50±0.47[a]	9.76±0.31[abc]	<0.001
AIP	-0.20±0.26	-0.31±0.20	-0.01±0.21[a]	-0.11±0.23[ab]	0.54±0.16[abc]	<0.001
BUN (mmol/L)	4.36±1.12	4.18±1.04	4.18±0.96	4.86±1.22[ab]	4.68±1.34[ab]	<0.001
SCr (umol/L)	58.54±9.33	58.16±8.64	56.86±8.68[a]	60.26±10.49[ab]	59.66±13.62	<0.001
eGFR (ml/min/1.73m2)	107.86±14.01	112.53±11.46	111.81±11.19	95.35±12.59[ab]	98.21±16.77[ab]	<0.001
ALT (U/L)	17.31±15.72	15.09±15.54	19.83±15.88[a]	20.24±14.76[a]	28.92±17.41[abc]	<0.001
AST (U/L)	22.21±8.36	20.92±7.58	21.65±8.19	25.09±9.14[ab]	28.24±9.82[ab]	<0.001
New-onset DM n, (%)	48 (1.2)	2 (0.1)	4 (0.7)	36 (3.5)	6 (7.1)	<0.001

Abbreviations: BMI, body mass index; SBP, systolic blood pressure; DBP, diastolic blood pressure; FBG, fasting blood glucose; TC, total cholesterol; TG, triglyceride; HDL-C, high-density lipoprotein cholesterol; LDL-C, low-density lipoprotein cholesterol; TyG, triglyceride-glucose inde；AIP, atherogenic index of plasma; BUN, blood urea nitrogen; Scr, creatinine; eGFR, estimated glomerular filtration rate; ALT, alanine transferase; AST, aspartate transferase; DM, diabetes mellitus.

TABLE S7. Multiple Cox proportional hazard regression analysis for DM incidence according to clusters by sex

Cluster	Males		Females	
	HR (95% CI)	*P*-value	HR (95% CI)	*P*-value
Cluster 1 vs Cluster 2	6.84 (2.06-22.76)	<0.001	6.44 (1.11-37.51)	0.038
Cluster 1 vs Cluster 3	22.35 (6.62-75.44)	<0.001	40.77 (8.22-202.08)	<0.001
Cluster 1 vs Cluster 4	29.10 (8.53-99.32)	<0.001	45.15 (6.47-315.35)	<0.001
Cluster 2 vs Cluster 3	3.27 (1.94-5.50)	<0.001	6.33 (1.83-21.87)	0.004
Cluster 2 vs Cluster 4	4.25 (2.59-6.99)	<0.001	7.01 (1.49-33.02)	0.014
Cluster 3 vs Cluster 4	1.30 (0.84-2.02)	0.238	1.11 (0.37-3.34)	0.856

Adjusted for SBP, DBP, TC, non-HDL, LDL, ALT, AST, BUN, Scr, eGFR, smoking status, drinking status, and family histroy of diabetes.
